# Medical Coordination Rescue Members’ and Ambulance Nurses’ Perspectives on a New Model for Mass Casualty and Disaster Management and a Novel Terror Attack Mitigation Approach in the Netherlands: A Qualitative Study

**DOI:** 10.1017/S1049023X21000790

**Published:** 2021-10

**Authors:** Sivera A.A. Berben, Lilian C.M. Vloet, Frans Lischer, Moniek Pieters, Johan de Cock

**Affiliations:** 1. Associate Professor, Emergency and Critical Care, Knowledge Center of Sustainable Healthcare, Institute of Nursing Studies, HAN University of Applied Sciences, Nijmegen, the Netherlands; Research Fellow, IQ Healthcare, Radboud University Medical Center, Nijmegen, the Netherlands; Senior Researcher, Academic Network of Applied Public Health and Emergency Medicine (ANAPHEM), Radboud University Medical Center, Nijmegen, the Netherlands; 2. Professor, Emergency and Critical Care, Knowledge Center of Sustainable Healthcare, Institute of Nursing Studies, HAN University of Applied Sciences, Nijmegen, the Netherlands; Research Fellow, IQ Healthcare, Radboud University Medical Center, Nijmegen, the Netherlands; Senior Researcher, Academic Network of Applied Public Health and Emergency Medicine (ANAPHEM), Radboud University Medical Center, Nijmegen, the Netherlands; 3. Network Coordinator, Academic Network of Applied Public Health and Emergency Medicine (ANAPHEM), Radboud University Medical Center, Nijmegen, the Netherlands; 4. Director of Public Health, Public Health Service Gelderland Zuid, Nijmegen, the Netherlands; 5. Senior Researcher, Scientific Coordinator, Academic Network of Applied Public Health and Emergency Medicine (ANAPHEM), Radboud University Medical Center, Nijmegen, the Netherlands

**Keywords:** CBRN, disaster medicine, EMS, mass casualty, MIMMS, terror

## Abstract

**Introduction:**

Mass-casualty incidents (MCIs), specifically incidents with chemical, biological, radiological, and nuclear agents (CBRN) or terrorist attacks, challenge medical coordination, rescue, availability, and adequate provision of prehospital and hospital-based emergency care. In the Netherlands, a new model for Mass Casualty and Disaster Management (MCDM) along with a Terror Attack Mitigation Approach (TAMA) was introduced in 2016.

**Study Objective:**

The objective of this study was to provide insight in the first experiences of health policy advisors and managers with a medical rescue coordinator and ambulance nursing background regarding the new MCDM and TAMA in order to identify strengths and pitfalls in emergency preparedness and to provide recommendations for improvement.

**Methods:**

The study had a qualitative design and was performed from January 2017 through June 2018. Purposeful sampling was used and the inclusion comprehended health policy advisors and managers with a medical rescue coordinator and ambulance nursing background involved in emergency preparedness. The respondents were interviewed semi-structured and the researchers used a topic list that was based on the literature and content of the newly introduced model and approach. All interviews were typed out verbatim and qualitative content analyzing was used in order to identify relevant themes.

**Results:**

Respondents based their perceptions on large-scale training exercises, as MCDM and TAMA were not yet used during MCIs. Perceived issues of MCDM were the two-tiered triage system, the change in focus from “stay and play” towards “scoop and run,” difficulties with new tasks and roles of professionals, and improvement in material provision. Regarding TAMA, all respondents supported the principles (do the most for the most; scoop and run; acceptable personal risk; never walk alone; and standard operational procedure); however, the definitions were lacking clarity while the awareness of optimal personal safety of professionals was absent.

As there are currently regional differences in the level of implementation of MCDM and TAMA, this may pose a risk for an optimal inter-regional collaboration.

**Conclusion:**

The conclusions refer to experiences of professionals in the Netherlands. Elements of the MCDM and TAMA were highly appreciated and seemed to improve emergency preparedness, while other aspects needed further attention, training, and integration in daily routine. The Netherlands’ MCDM model and TAMA will need continuous systematic evaluation based on (inter)national performance criteria in order to underpin the useful and effective elements and to improve the observed pitfalls in emergency preparedness.

## Introduction

(Pre)hospital emergency care provision of ambulance professionals and medical coordination rescue members during mass-casualty incidents (MCIs) differs considerably from their routine daily assistance to patients with life-threatening illness or injuries. In addition to logistic challenges and taking care of multiple patients, they have to deal with potential chaos, a large-scale coordination and management approach, with less control and stressful working conditions. Personnel of Emergency Medical Services (EMS) and medical coordination rescue members need to be skilled and prepared for their work during MCIs.^1–3^

**Figure 1.**
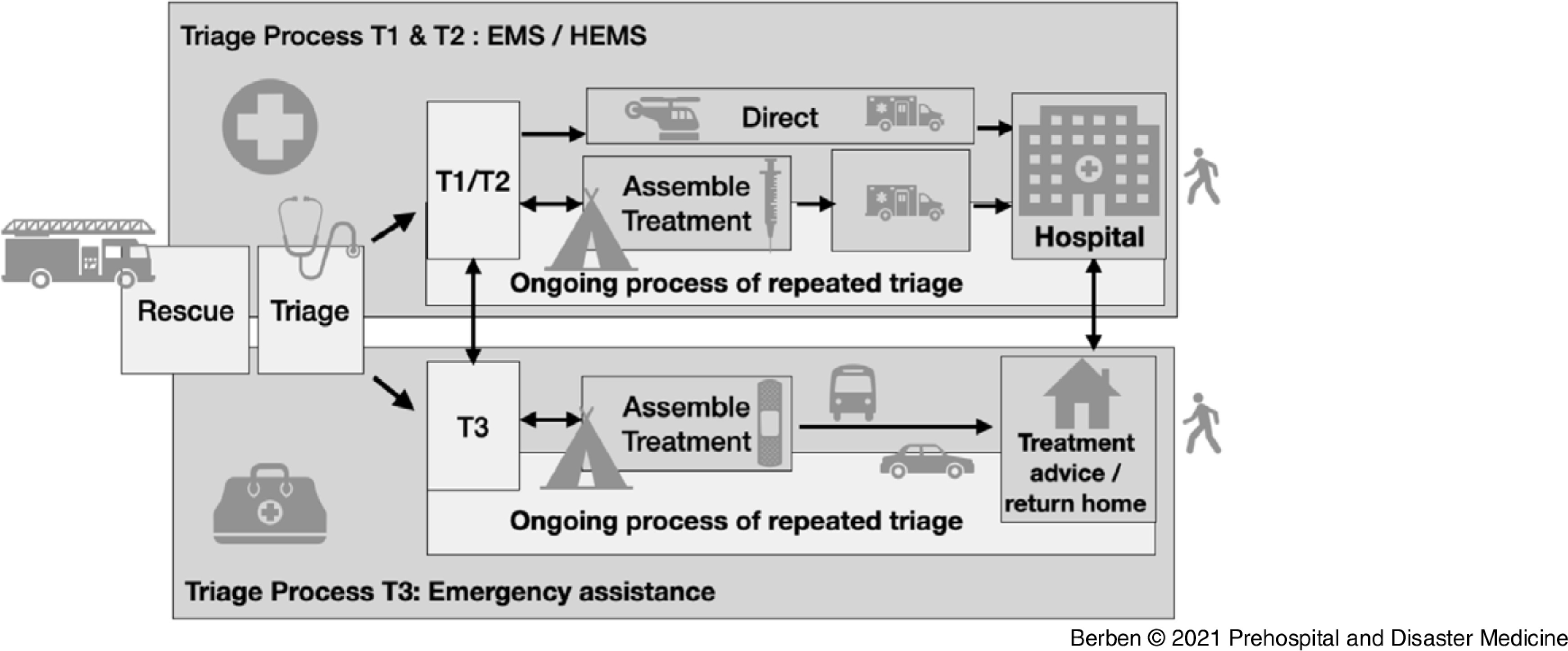
Netherlands MCDM Model for Emergency Preparedness. Abbreviations: EMS, Emergency Medical Services; HEMS, helicopter Emergency Medical Services; MCDM, Mass Casualty and Disaster Management.

In order to adequately organize EMS resources and their response during MCIs, a new national approach for Mass Casualty and Disaster Management (MCDM) was released in the Netherlands (Figure 1).^4,5^ Ideally, once an MCI has been identified, a well-coordinated flow of events will occur using three phases: triage, treatment, and transportation.^6^

The MCDM approach is characterized by a two-tiered triage system and dispersion of basic and advanced (pre)hospital emergency care resources based on the patients’ triage level with the Major Incident Medical Management and Support priority classification.^7^ Patients with triage levels T1 and T2 are assembled, treated, and transported by large-scale (helicopter) EMS (HEMS). These services consist of nursing-staffed ambulances supported by physician-staffed HEMS. However, T3 victims are assembled and treated by regional lay-staffed emergency response teams, employed with Red Cross (The Netherlands Red Cross; The Hague, The Netherlands) volunteers.

Additional logistic resources in the model are supplied by the Dutch Institute for Safety (IFV; Arnhem/Zoetermeer, The Netherlands). Direction and medical coordination are provided by managers and health policy advisors of the Regional Public Health Medical Emergency Planning and Preparedness Organization (GGD GHOR; Utrecht, The Netherlands), often with an ambulance nursing or medical rescue member background.

Mass Casualty and Disaster Management is based on the existing regular day-to-day capacity. What’s new is that capacity can be further increased by deploying roster-free ambulance staff (professionals) and trained volunteers of the Red Cross. The introduction of two (instead of one) main triage flow(s) lead to adaptations in the spatial distribution of human resources (tasks and roles), equipment, logistics, plans, and procedures. Therefore, the new model affects all the consecutive basic steps in the chain (Figure 1).

Simultaneous with the introduction of MCDM, at the end of 2016, the risk of potential terrorist attacks was high, and therefore six out of the 25 regional Safety Authorities prepared a Terror Attack Mitigation Approach (TAMA). The aim was to combat the effects of terror attacks in the Netherlands and to assist professionals with clear points of departure in their approach. This approach is based on five principles and measures: (1) Do the most for the most; (2) Scoop and run; (3) Acceptable risk for health care providers; (4) Never walk alone; and (5) Standard operational procedures (Table 1). Point four “Never walk alone” refers to the necessity of supra-regional and national assistance in any case of TAMA.

**Table 1.** The Five TAMA Principles and Measures

1	Do the Most for the Most	The aim is to achieve the highest possible survival rate for the group of victims.
2	Scoop and Run	Casualty triage outside the direct area of the incident for as many as possible and transportation to the nearest possible hospital.
3	Acceptable Risk for Health Care Providers	During the attack, a safe working environment cannot be guaranteed. An acceptable risk will be pursued to protect health care providers as well as possible.
4	Never Walk Alone	Consequence control needs proper neighborly assistance, national deployment, and assistance.
5	Standard Operational Procedure	Procedures for medical assistance should match best with regular day-to-day practice.

Abbreviation: TAMA, Terror Attack Mitigation Approach.

Where MCDM is a guideline for large-scale incidents, TAMA is a specific approach describing principles and measures to be taken during a terror attack.^8^ In 2016, TAMA was not (yet) integrated in the MCDM model. As the MCDM and TAMA both involved a radical change for the regional medical rescue coordinators and the ambulance nurses, the objective of this study was to gain insight in the first experiences of health policy advisors and managers with a medical rescue coordinator and ambulance nursing background with MCDM and TAMA. The primary objective was to identify strengths and pitfalls in (pre)hospital emergency preparedness under (potential) MCI conditions and to provide recommendations for improvement.

## Methods

### Study Design

The qualitative study was performed from January 2017 through June 2018. The outcome variable was the respondents’ opinion on usefulness and clarity of the new approach and their first experiences and perceived strengths and pitfalls. Through in-depth qualitative interviews, new meanings and appreciations can be developed.^9^ Contextual and explanatory information from key performance actors (health policy advisors and managers with an ambulance nursing and medical rescue coordinator background) can support further development and implementation of (pre)hospital emergency preparedness.^10^

The Ethics Review Board of HAN University of Applied Sciences, Nijmegen, the Netherlands waived the need for review of the study, as respondents were not exposed to any intervention nor were the interviews intimidating (Protocol ID number ECO 250.03/21). The Consolidated Criteria for Reporting Qualitative Research (COREQ) checklist was used to guide the reporting of this study.^11^

### Selection of Participants

A two-tiered inclusion process was used, as the interviews regarding the MCDM model (January-June 2017) were subsequently followed by interviews regarding TAMA (January-June 2018). First, the senior researchers (JdC, SB) purposefully sampled respondents for this study of five (out of 25) Safety Authorities (each region includes a regional EMS and a Regional Public Health Medical Emergency Planning and Preparedness Organization). The regional Safety Authority and the participants needed to have (ample) experience with MDCM during MCIs or training exercises. Regional Safety Authorities and participants who were not yet using the new model and approach were excluded. In the second part of the study, the researchers purposefully sampled respondents of five regional Safety Authorities of which two out of five were not engaged in the development of TAMA. Respondents’ characteristics are described in Table 2. All respondents provided informed consent.

**Table 2.** Overview of Participants

Participant	Study	Region	Years of Experience	Educational Background	Picket Function	Position
1	MCDM	North	3	Crisis and Public Order Management	General Commander of Medical Care	Health Policy Advisor
2	MCDM	National	17	Nurse	*Not Applicable*	Health Policy Advisor
3	MCDM/TAMA	South	12	EMS Nurse	General Commander of Medical Care	Specialist Incident Response
4	MCDM/TAMA	East	11	EMS Nurse	General Commander of Medical Care	Head of Regional MEPPO
5	MCDM/TAMA	South	18	Sociologist	General Commander of Medical Care	Head of Regional MEPPO
6	MCDM/TAMA	West	16	Law/Economics	General Commander of Medical Care	Head of Regional MEPPO
7	MCDM/TAMA	West	20	Ambulance EMS Nurse	Medical Officer Major Incident	Team Leader/Major Incident Medical Officer
8	MCDM	West	9	Ambulance EMS Nurse	Head Operational Center	Health Policy Advisor
9	MCDM	West	17	Ambulance EMS Nurse	Medical Officer Major Incident	Regional Training Coordinator EMS Ambulance
10	MCDM	North	4	Law	General Commander of Medical Care	Head of Regional MEPPO
11	MCDM	East	10	Environmental Sciences	Head of Information for Medical Care	Health Policy Advisor
12	MCDM	National	17	Social Pedagogy	*Not Applicable*	Health Policy Advisor
13	TAMA	West	12	Public Health Nurse	General Commander of Medical Care	Project Leader TAMA
14	TAMA	East	20	Ambulance EMS Nurse	Medical Officer Major Incident	Regional Training Coordinator EMS Ambulance
15	TAMA	National	13	Ambulance EMS Nurse	Medical Officer Major Incident	Liaison MEPPO
16	TAMA	National	16	Business Administration	*Not Applicable*	Health Policy Advisor TAMA
17	TAMA	West	8	Ambulance EMS Nurse	Medical Officer Major Incident	Health Policy Advisor
18	TAMA	West	2	Business Administration	*Not Applicable*	Secretary Project team TAMA

Abbreviations: EMS, Emergency Medical Services; MCDM, Mass Casualty and Disaster Management; MEPPO, Medical Preparedness and Planning Office; TAMA, Terror Attack Mitigation Approach.

### Data Collection and Processing

Respondents were interviewed in a quiet room located in or nearby their offices and no other persons were present. The research team consisted of five research assistants - bachelor students of medical assistants in their last year of education (MP, KS, JR, JS, PO) - and two senior research members (JdC, SB). The latter had ample experience in performance and supervision of qualitative interviews. During all the interviews, at least one senior researcher was present. A semi-structured topic list was used, and the interview guide was based on the literature and content of MCDM or TAMA. The research students followed a specific interview training before the start of the qualitative interviews. The topic lists for MCDM and TAMA were pilot tested in an interview. Based on the pilot tests, the sequence of the questions was changed and some questions were slightly changed in order to enhance the openness of the respondents. The research students introduced the study and started with the 60- to 90-minute interview. The senior researcher provided hands-on supervision and subsequently asked more in-depth questions to achieve enrichment and saturation of the data. The interviews were audio recorded and field notes were taken.

### Data Analysis

All interviews were typed out verbatim based on the audio records. Furthermore, the field notes were captured. The senior researchers (JdC, SB) together with two research assistants (MP, KS) analyzed the data on experiences related to the Netherlands MCDM model. The data on TAMA were analyzed together with the other group of research assistants (JR, JS, PO). For both topics, a specific coding tree was developed and themes were derived from the data.^12^ Data analysis was performed through the long table method with the use of post-it notes, afterward supported by Microsoft (Microsoft Corp.; Redmond, Washington USA) applications in Word (version 16.46) and Excel (version 16.46), as the research students had no access to qualitative data analysis software such as Atlas-ti (ATLAS.ti Scientific Software Development GmbH; Berlin, Berlin).

## Results

The respondents reflected on the general principles and characteristics of MCDM and TAMA, described the bottle necks, and reflected on barriers and facilitators of the implementation. Table 3 summarizes the issues described by the respondents as present (“+”) or not present (“–”).

**Table 3.** Respondent Perspectives on the MCDM Model and TAMA

	MCDM	TAMA
**1. General Principles and Characteristics**		
- Real-Time Experience versus Exercises	+	+
- Triage	+	–
- Scoop and Run	+	+
- Competences/New Tasks and Roles	+	+
- Materials	+	–
- Relation between MCDM and TAMA	–	+
- Five Principles	–	+
**2. Implementation**		
- Introduction	+	+
- Dissemination	+	+
- Education	+	–
- Training and Exercises	+	–
- Collaboration	+	+

Abbreviations: MCDM, Mass Casualty and Disaster Management; TAMA, Terror Attack Mitigation Approach.

### General Principles and Characteristics – MCDM

*Real-Time Experience versus Exercises—*Experience with the new MCDM model was primarily gained from multi-disciplinary MCI training exercises. Some respondents indicated that MCDM was primarily based on assumptions rather than evidence. The new model was perceived as a prescribed and strict plan, rather than a guide for adequate emergency preparedness in case of MCIs. On the other hand, the respondents positively experienced the flexibility of the model to suit various types of incidents (eg, ranging from major fire to road accidents). On a regional level, some health policy advisors and staff gave their own twist to the model, which drew criticism among some other respondents and increased their opinion of the risk for a hampering safety approach on inter-regional and national level:

What I think about it in general is that MCDM is based too much on assumptions. These are not always properly tested and realistic. [Interview 5]

*Triage/Scoop and Run—*The respondents regarded adequate triage as an important and basic intervention in MCDM. They experienced the procedure and the triage protocol for proper communication as complex, and furthermore mentioned that supporting tools such as triage cards were not used adequately during training. Respondents generally were in favor of the scoop and run focus, and they believed it would result in better (pre)hospital emergency health care. Scoop and run was seen as a clear, straightforward, and simple approach which helped them to operate easily under complex circumstances:

Yes, the purpose of the model contributes to better patient care when you consider the quality of the upscaled care. And why? Because the terminology is uniform, the approach is uniform nationally, and the tasks are assigned correctly. [Interview 6]

*Competences/New Task and Roles—*The introduction of a task card to enhance the medical coordination rescue members and ambulance nurses in performing their new tasks and roles was appreciated. Respondents mentioned that the task card needed further practice experience to work with it effectively. Some health care providers, such as the first ambulance nurse on scene, felt insecure in the “command and control” role. Respondents also mentioned difficulties in the performance of the role of the dispatch center in “command and control” in case of chaos in the field, due to complex communication. They considered it a major issue to require competencies and to stay competent for new tasks and roles in the MCDM model. On a regional level, some indicated that they invested in more (table-top) exercises to improve this shortcoming:

It is because we have been indoctrinated by a model that we have been familiar with for over fifteen years. And now at once, you have to let go and you have to start thinking in a different pattern. That is difficult. [Interview 5]

And the tricky part is that you have to maintain it [education]. So, it is not enough to train once and then think “it is all right.” You should do that every year. [Interview 3]

The changing role and deployment of the Red Cross volunteers was considered a positive development, while others thought that it required a lot of effort from the Red Cross. At the time of the interview, it was unknown to the respondents whether these requirements could be met by the Red Cross.

*Materials—*It seemed impossible to have enough materials and vehicles at the right location in time during the regional multi-disciplinary MCI exercises. At the same time, respondents stated that a proper startup on scene was considered to be crucial for the further course of emergency assistance in case of MCI. The sturdy tents for the stay and play focus in the old model had been replaced by smaller and less well-equipped tents with the new focus of scoop and run in mind. Some respondents felt that these tents were unsafe for adequate triage.

A positive change in the new model was that materials were sourced from the regular ambulance EMS inventory rather than from a specific MCI inventory. This was seen as a cost-effective development. Respondents experienced that professionals were clearly provided with more up-to-date materials:

… in the past, we had a container literally completely filled up with materials. That was getting out of date all the time and had to be replaced regularly (eg, medication, oxygen, water, and all). So, a lot of things were thrown away all the time. Now it is part of the regular stock, so financially it is an improvement. [Interview 1]

### General Principles and Characteristics – TAMA

Some respondents saw TAMA as a specific scenario within the MCDM model, especially with regard to the personal safety of prehospital ambulance nurses and medical rescue coordinators and the specific zone classification (hot, warm, cold) used for chemical, biological, radiological, and nuclear agent (CBRN) purposes. In general, there was limited experience with the new approach. Respondents saw MCDM as a scenario-independent model. Other respondents saw TAMA as an approach on its own in case of terrorist attacks, to be applied in the hot and warm zone. Several respondents preferred the term “extreme violence approach” instead of TAMA, because a potential terrorist motive for violence attacks usually becomes classified terrorist motive at a later stage of the EMS process, or even afterwards. According to some respondents, TAMA requires a change in the way of thinking and acting. They advised that the focus of TAMA should be on urgent transport of victims from the hot zone into the cold zone with only a short urgent treatment on scene.

*Five Principles—*According to the respondents, the TAMA principles “scoop and run” and “acceptable risk” were the most prominent issues in the novel approach. Although respondents found that the basic principles were clearly defined, it appeared during the interviews that they interpreted them differently. The respondents saw the basic principle of “do the most for the most” as a change in priority towards a large number of survivors rather than a priority towards the optimal survival of the individual patient. Practical experience in this new and different way of decision making was lacking.

The opinion of respondents on “acceptable risk” differed considerably. Some placed the responsibility of acceptable risk on the professionals themselves, because they independently decided whether or not to enter the warm zone. Other respondents wondered whether their colleagues were sufficiently aware that the TAMA approach itself did not guarantee personal safety of emergency professionals on scene. Time for and priority on personal safety on scene was experienced not to be in line with the professional drive of professionals. The respondents sensed that personal insecurity was the main issue to deal with:

If you look at it realistically, you realize that it does not only is about terrorist attacks, but also extreme violent incidents. [Interview 2]

Acceptable risk is when you can’t 100 percent guarantee that something is safe, but as long as the police state it is safe, we’ll trust it. [Interview 2]

With TAMA, we have agreed that safety is relative to some degree if the task is to save peoples’ lives. [Interview 3]

### Implementation

*MCDM—*MCDM was introduced and disseminated through the national and regional structure of the Safety Authorities. Although the dissemination was efficient, the implementation of MCDM by a position paper and e-learning was hampered due to lack of priority in the regions. Furthermore, e-learning was not tailored to the needs of the professionals regarding their new roles. Also, the high workload in ambulance EMS and other multi-disciplinary partners, such as the police and fire brigade, obstructed the introduction. However, a positive attitude of health policy advisors involved in the (regional) implementation positively contributed to acceptance and use of the model.

A one-time multi-disciplinary exercise training was perceived as insufficient to improve knowledge and competences of responsible professionals, both on scene and in the chain of emergency care. The limited budget for staff, lotus victims, vehicles and equipment, and a suitable location for the large-scale exercise training required smarter solutions according to the respondents:

Look, you use that e-learning every now and then to freshen up a bit, but to start working with a completely new concept, is [e-learning] too meager an instrument? [Interview 2, line number 191-192]

The problem is that we used to know what quality we had, because we provided the training ourselves. The training is now entirely with the Red Cross. I have no idea what they are teaching those people there. [Interview 5, line number 619-621]

*TAMA—*TAMA had been introduced and disseminated to other regions through a position paper and a power point presentation (Microsoft Corp.; Redmond, Washington USA). In contrast to MCDM, TAMA did not gain a mandatory status in the regional Safety Authorities. However, all the regions responded positively to the provision of the TAMA approach. Respondents suggested that regions previously experienced a gap in guidance in emergency preparedness, specifically for CBRN and terrorist attacks.

Each region implemented TAMA in its own way and decided how to prepare for a terrorist attack. All the respondents shared the opinion that there should be national consensus on uniformity within the mass-casualty chain, as to date, central management and action frameworks are sometimes lacking:

The ambulance service trained in a way that we will only act when it is safe to do so. That is something that does not go together with a terrorist attack. [Interview 5, lines 28-29]

All parties are working in their own regions in their own way. Regions attempt to coordinate in a uniform way, which is extremely difficult to achieve. [Interview 1]

### Integration of MCDM and TAMA

Based on multi-disciplinary exercises, all respondents agreed that a main point for improvement was an integration of MCDM and TAMA, and an increased collaboration between the different emergency services (eg, ambulance EMS, police, and fire workers) in the region and on an inter-regional level. The need for more collaboration also included coordination of working methods and materials in use during MCIs.

## Discussion

Respondent’s perceptions on general principles and characteristics of MCDM and TAMA were mainly based on experiences with limited multi-disciplinary MCI exercises. This study showed a number of improvements of the new MCDM compared to the former large-scale model of emergency preparedness. These included the possibilities for upscaling of staff, the two-tiered triage system with scoop and run principles for T1-T2 victims, and the provision of up-to-date materials by ambulance EMS and IFV. Bottlenecks in the new model were the training and maintenance of skills and competencies of professionals for new roles, while insight in competences of lay-staff of the Red Cross was lacking. The underlying theoretical principles of MCDM based on the literature were not clearly explained at the introduction. In TAMA, the principles “scoop and run” and “acceptable risk,” and furthermore attention for personal safety, were seen as important improvements.

The principle of “acceptable risk” is a sensitive subject to which various responses were received during the interviews. Some respondents placed this responsibility primarily with the first responders and ambulance nurses themselves. Others indicated that they wondered whether the care providers were aware that safety cannot be guaranteed. In addition, other respondents believed that feeling safe is one of the major issues to be dealt with. The interviews showed that more effort should be put into incorporating knowledge described in the literature since the United States 9/11 attacks in 2001 in the multi-disciplinary education and training emergency preparedness programs of ambulance nurses and rescue members in the chain of emergency care.^13^

In reflection to the change from scoop and run versus the stay and play model, the literature describes relevant differences in effectiveness of these approaches.^14^ However, data to substantiate the effectiveness of scoop and run in the Netherlands are lacking and the underpinning of the MCDM model was primarily based on consensus among professionals. Therefore, the optimal model for large-scale MCI management in the literature couldn’t be identified yet and should be studied based on future systematic evaluations.

Also, in case of an MCI incident, capacity aspects of MCI management, in relation to the number of victims, the scale of the MCI, environmental characteristics, distance to the nearby trauma center, the medical capacity in terms of hospital beds and ambulances, and (pre)hospital staff competencies, might be relevant on the choice for the preferred model.^15,16^ However, there is large consensus that prehospital treatment and transfer time of victims should be as short as possible, in favor of scoop and run as the standard approach.^17^ This approach is only feasible as long as the medical chain can handle the influx of casualties.^18^

As MCDM and TAMA deviate on a number of essential characteristics from daily practice of EMS and the previous approach for MCI conditions, the introduction, implementation, and familiarization of a new model and novel approach needs proper, timely, and extensive attention. Especially for medical coordinator rescue members, the learning of new roles and tasks should not be under-estimated as it also will affect the seamless embedding with multi-disciplinary roles, tasks, and responsibilities. Furthermore, current regional differences in implementation of MCDM and TAMA in regional Safety Authorities potentially complicate and compromise multi-disciplinary inter-regional collaboration during MCIs or terrorist attacks.

Both MCDM and TAMA were introduced in the regional Safety Authorities while the reasoning behind the model and approach were not clearly explicated or linked to the literature or best practices. This lack of insight substantially hampered the implementation according to the respondents. Implementation models describe that effective implementation requires a thorough analysis of the target group (involvement) and characteristics and the fit of the intervention into current practice,^19^ followed by a tailored development of an implementation strategy, where it is known that only the use of information and education strategies is ineffective.^20^

It seemed that the bottom-up approach and introduction of TAMA generally led to a better support and acceptance among professionals than the nation-wide top-down introduction and implementation of MCDM. However, the introduction of five points of departure of TAMA led to different interpretations between respondents. Next to the unclear status of MCDM and TAMA, criteria for evaluation and inspection during MCIs were lacking and the intended coherence of the MCDM and TAMA was not made explicit.

Therefore, the introduction and implementation of new (nation-wide) MCDM model and TAMA need a systematic and stepwise follow-up.

Also, a systematic nation-wide planning and evaluation of MCI exercises is needed. This will lead to an evidence-based development of prehospital emergency preparedness. Parties within the medical emergency chain, as well as multi-disciplinary partners, should be involved in this systematic evaluation process, including international experiences.

## Study Limitations

This study has some limitations. Due to the qualitative approach chosen, insight in the first perceptions of purposeful sampled respondents was mainly based on MCI exercises and not real-time MCIs as real-time MCIs tend to have relatively low frequencies of occurrence as compared to day-to-day acute care. Although saturation was reached in the data, possibly the sample did not provide a full insight in experiences with the new model and approach. However, the multi-disciplinary training sessions provided a valuable first insight in strengths and pitfalls in the implementation and use of MCDM and TAMA.

Another limitation was the purposeful selected sample, although the included respondents represented different geographical regions in the Netherlands and data saturation was reached within the sample.

Possibly, a future quantitative follow-up evaluation might give insight in the frequency and impact of identified perceptions in this study. If the study was performed a longer time after the implementation, additional issues could have been identified. Therefore, systematic and frequent evaluation of MCI management is recommended in order to develop evidence-based knowledge and methods for prehospital MCI management, including CBRN conditions. Development of (inter)national evaluation criteria for MCI management therefore would be helpful.

## Conclusions

The conclusions refer to experiences with MCDM and TAMA of professionals in the Netherlands, and were mainly based on emergency preparedness exercises and training sessions. Several elements were highly appreciated, while other aspects needed further development, attention, training, and integration in daily routine. Both the MCDM model and TAMA will need a more tailored implementation and continuous systematic evaluation based on (inter)national criteria in order to underpin the theoretical principles and effective elements for emergency preparedness, and furthermore to improve the observed pitfalls.
